# Quenched Residual Stress Reduction in Pentagon-Curved Aluminum Alloy Forgings Using the Bulging Process

**DOI:** 10.3390/ma16175910

**Published:** 2023-08-29

**Authors:** Chuanwei Luo, Chen Li, Xinquan Zhang, Yunxin Wu, Tao Zhang

**Affiliations:** 1 School of Mechanical and Electrical Engineering, Central South University, Changsha 410083, China; 193701027@csu.edu.cn; 2 State Key Laboratory of High-Performance Complex Manufacturing, Central South University, Changsha 410083, China; 3 Avic the First Aircraft Institute, Xi’an 710000, China; lichen_1983_2002@163.com (C.L.); xinquan417@126.com (X.Z.); 4 Light Alloy Research Institute, Central South University, Changsha 410083, China

**Keywords:** pentagon-curved forging, quenched residual stress, bulging, overall stress reduction assessment

## Abstract

Quenched residual stress in pentagon-curved forgings (PCGs) often leads to severe deformation during subsequent machining operations. This study aims to mitigate the quenched residual stress in PCGs through the implementation of the bulging method. The edge distance ratio (e/D), a geometric characteristic of PCGs, is defined and considered in the established thermo-mechanical model, which incorporates the effects of quenched residual stress. Increasing e/D resulted in amplified maximum internal stresses and surface stresses. To address this issue, a bulging finite element (FE) model was developed to effectively alleviate the quenched residual stress. The stress reduction in surface stress and internal stress was qualified using average stress reduction ($R_{a}$) and peak stress reduction ($R_{p}$), respectively. Notably, stress reduction exhibited an inverse relationship with e/D, indicating that decreasing e/D yields greater stress reduction. Furthermore, an overall stress reduction assessment was conducted for different bulging ratios, revealing that the stress reduction increased as the bulging ratio increased. A comprehensive comparison of different bulging ratios highlighted 2% as the most optimal bulging ratio for stress reduction in PCGs. X-ray diffraction measurement and the contour method were employed to determine surface stress and internal stress, respectively. The experimental results were in agreement with the simulation outcomes, validating the high accuracy of the FE model.

## 1. Introduction

The increasing demand for lightweight materials in the aerospace, automotive, and transport industries, driven by global energy shortages, has led to the widespread use of 7000-series Al alloys in aircraft monolithic structural components. These alloys offer benefits such as reduced consumption, improved production efficiency, lower manufacturing costs, and enhanced service performance [1,2,3,4,5,6]. Typically, 7050 aluminum alloys undergo a quenching process to achieve a supersaturated solid solution before undergoing aging heat treatment to enhance their mechanical properties [7,8]. However, to ensure optimal material performance after quenching, a high cooling rate is necessary due to the quenching sensitivity, resulting in a significant thermal gradient that induces substantial residual stress [9,10]. The distortion related to residual stress poses a significant challenge during the machining process, making the prediction and control of residual stress crucial for production purposes. Numerous studies have been conducted on the stress evolution during the quenching process. Bouissa [11] and Koc et al. [12] have established thermo-mechanical models based on finite element analysis (FEM) to predict the residual stress induced during the quenching process. Zhang [13] studied the relationship between residual stress and material parameters (thermal expansion coefficients, elastic moduli, and yield strengths) for a 2A14 Al alloy, and investigated stress evolution during quenching. The Sandia National Laboratory [14,15] in the US has conducted research on aerospace-grade aluminum alloys, revealing that the magnitude of residual stress resulting from quenching is approximately linearly proportional to the thickness of the plate [16,17]. These studies highlight that quenched stress is influenced by the thermal gradient and limited by the as-quenched yield strength. The ability to predict and control the magnitude and distribution of quenching residual stress holds significant engineering importance for the manufacturing industry.

Although it is impossible to entirely avoid residual stresses, their extent can be reduced through a series of post-processes. One commonly employed method is mechanical stress leveling, which effectively reduces quenched residual stress. Compression and stretching models have been established for wrought Al alloys, demonstrating significant stress reduction capabilities. However, increasing the percentage deformation beyond 3% does not yield additional advantages [18]. Liu [19] conducted research on the quenched stress reduction of a 7050 aluminum alloy using cold compression, employing the contour method and neutron diffraction to determine the redistribution of residual stress. Both methods exhibited consistent trends and magnitudes of residual stress. Koc [12] developed FEM models to simulate the quenching and pre-stretching/compression processes of a 7050 aluminum alloy, obtaining the relationship between stress relief and strain.

The application of bulging primarily focuses on part forming, while limited attention has been given to stress reduction during the bulging process. MR [20] established a 2D FEM model to explore how the bulging ratio and edge distance ratio impact the residual stress distribution around a hole. Their findings emphasized the significant influence of the edge distance ratio on residual stress. Zhang [21] presented a closed-form elastic-plastic solution that accurately assesses the distribution of residual stress around a cold-expanded fastener hole. Wei [22] investigated the effect of the bulging process on residual stress in TC4 Alloy Ring forgings, demonstrating stress reduction and improved uniformity in residual stress distribution resulting from the bulging process. Li [23] employed FEM to study the impact of bulging on quenched residual stress, examining various bulging ratios to evaluate stress reduction. The results revealed a decrease in residual stress with an increasing bulging ratio. 

Pentagon-curved forgings (PCGs) serve as integral blank materials for manufacturing aircraft window frames. During the subsequent machining process, approximately 90% of the material is removed. However, after the quenching stage, these forgings experience significant residual stress, leading to substantial deformation during the machining. Even minor deformations require corrective measures, while severe deformations may result in part failure. These issues not only escalate processing costs, but also impair production efficiency. To minimize PCG deformation during machining, it is imperative to reduce residual stress following quenching. The irregular geometric shape of PCGs prevents the application of stretching for cold deformation. The cold compression method, on the other hand, faces limitations in hydraulic loads and requires multiple steps, resulting in prolonged processing times. Bulging, a cold deformation method combining stretching and compression, offers advantages for workpieces with center holes. Therefore, this study aims to investigate stress reduction in pentagon-curved forgings using the bulging method. FEMs representing the quenching and quenching–bulging processes for PCGs are established. Subsequently, an overall stress reduction assessment for the PCGs is developed using the weighted sum method. Finally, experiments are conducted to validate the accuracy of the simulation models.

## 2. Models Description

A PCG is made using a 7050 aluminum alloy forging plate. The workpiece possesses a three-dimensional size of 310 mm × 240 mm × 50 mm, with a strategically positioned hole of diameter ϕ100 intended for subsequent processes. In different sections, this component exhibits varying curvatures ranging from 0.002 to 0.005. 

**Figure 1:**
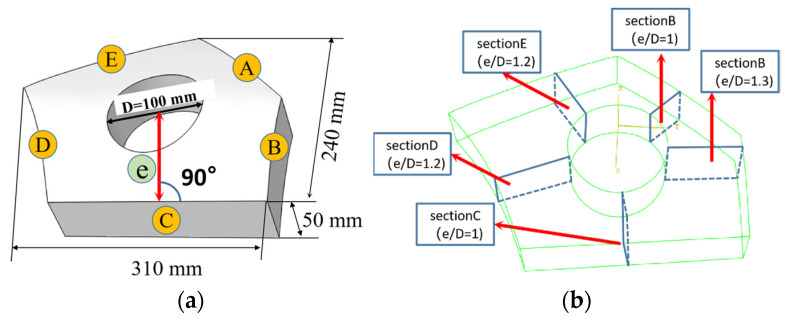
CAD model of The PCG and its three dimensions. (**a**) The three-dimensional PCG; (**b**) geometric feature classification.

The part displays an asymmetric geometry, and its geometric features are characterized by the edge distance ratio (e/D). The edge distance ratio is determined by measuring the verticals distance (e) from the center of the hole to each free edge, and dividing it by the diameter of the hole (D). To facilitate understanding, Table 1 provides a comprehensive list of the e/D values associated with the PCG, which can be further approximated and categorized into three distinct sections, as illustrated in Figure 1. 

### 2.1. Quenching Model

The quenching process was simulated using the thermal–mechanical coupling model in the commercial FE software ABAQUS (version 6.14). Quenching is a non-linear problem that contains thermal, plastic, elastic, and phase transformation phenomena. However, it is important to note that no phase transformation occurs in the 7050 aluminum alloy during the quenching process. The simulation utilized a C3D8RT mesh type with a size of 5 mm × 5 mm × 5 mm. The initial temperature of the PCG before quenching was 477 °C. Subsequently, the workpiece was immersed in water at a temperature of 20 °C. The entire immersion process took about 15 min. The material parameters for the 7050 aluminum alloy, necessary for the simulation, were obtained from experiments and are presented in Table 2. It should be noted that all parameters are input in the matrix form into the ABAQUS (version 6.14) software for calculation. The transfer coefficient between the part and water was set to 10,000 W/m^2^·K. To minimize the exposure time of the part to air, an axial water quenching method was adopted. Therefore, the FEM quenching process consisted of two steps. The first step involved the immersion process with a step time of 0.5 s. The second step was the actual quenching process, which lasted for 900 s.

### 2.2. Bulging Model

To mitigate the residual stress resulting from quenching, a bulging technique was implemented for the PCG. The underlying principle of the bulging process comprises an upper die, a lower die, a core shaft, and bulging blocks. The bulging procedure can be described as follows: (a) The workpiece was securely positioned on the lower die. (b) Bulging blocks were carefully inserted into the central hole of the workpiece. (c) The core shaft was then inserted into bulging blocks. (d) Utilizing hydraulic force, the core shaft was propelled downward, thereby inducing axial movement. Consequently, the bulging blocks underwent radial displacement, leading to plastic deformation of the workpiece. 

**Figure 2:**
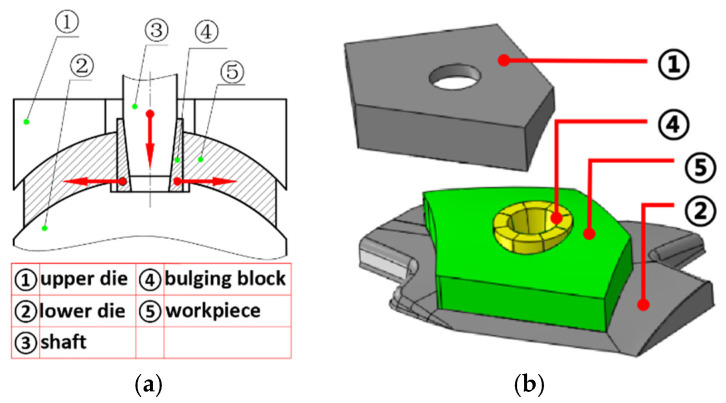
Bulging process and the three-dimensional FEM model. (**a**) Bulging process; (**b**) FEM model. The red arrow in subfigure (**a**) indicates the direction of movement.

In the simulation process, all fixtures were modeled as rigid bodies. The interaction between the fixtures and the PCG was defined as a general contact, incorporating a tangential behavior friction coefficient of 0.15. This friction coefficient was determined using the plate method, which involved calculating the ratio between the pulling force in the direction of motion and the force perpendicular to the motion under uniform conditions. In the FE model, the interaction and movement between the fixtures had no effect on the analysis of deformation and stress in the PCG. As a result, the axial movement of the shaft could be ignored, and the bulging process is directly accomplished through the radial displacement of the bulging blocks. The bulging FE model is depicted in Figure 2b.

For the bulging process, the flow stress was applied with a strain rate of 0.01/s, consistent with the strain rate encountered during cold work. The stress–strain data, required as an input for the finite model, were obtained from a room-temperature tensile test and input in the matrix form. The literature suggests that residual stress in aluminum alloys can be reduced by 1–4% through stretching or compression [24]. In this study, the bulging ratio was set at 1%, 2%, and 3%. The bulging ratio is obtained using Equation (1).

(1) $$CE=\frac{D_{1}-D_{0}}{D_{0}}\times 100\%$$

Note: *D*_1_ represents the diameter after bulging. *D*_0_ denotes the diameter before bulging.

## 3. Results and Discussion

### 3.1. Quenching Residual Stress

#### 3.1.1. Overall Residual Stress Distribution

**Figure 3:**
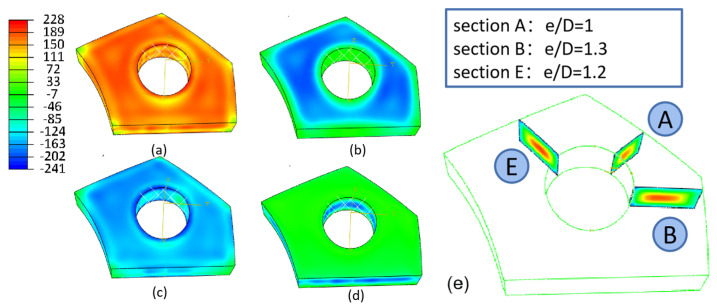
Contours of residual stress distribution on the quenched forging. (**a**) Mises stress; (**b**) radial stress; (**c**) hoop stress; (**d**) axial stress; (**e**) internal hoop stress.

Figure 3 illustrates the stress distribution map following the quenching process. The surface exhibits a compressive residual stress distribution, while tensile stresses are observed at the center. The magnitudes of compressive stress reach a maximum of approximately −240 MPa at the hole edge, which corresponds to about 80% of the yield stress (290 MPa) of the quenched 7050 aluminum alloy. Along the path from the hole edge, a radial-component stress of 220 MPa is generated. Comparing the hoop and radial component stresses with the Mises stress, it is evident that the hoop component stress has a great impact on the magnitude of the Mises stress compared to the radial stress. In addition, the stress levels at each corner of the PCG are relatively lower on the surface. These corners, composed of three surfaces, experience a higher cooling rate, leading to increased residual stress that may surpass the yield stress. Consequently, these corners undergo deformation, resulting in stress release. As a result, the residual stress induced at the free edge is larger than that at the corners, but smaller than on the majority of the surfaces in most cases [25]. The magnitude of the axial component stress is lower than that of the radial and hoop components. It is most pronounced on surfaces that are parallel to the axis orientation.

**Figure 4:**
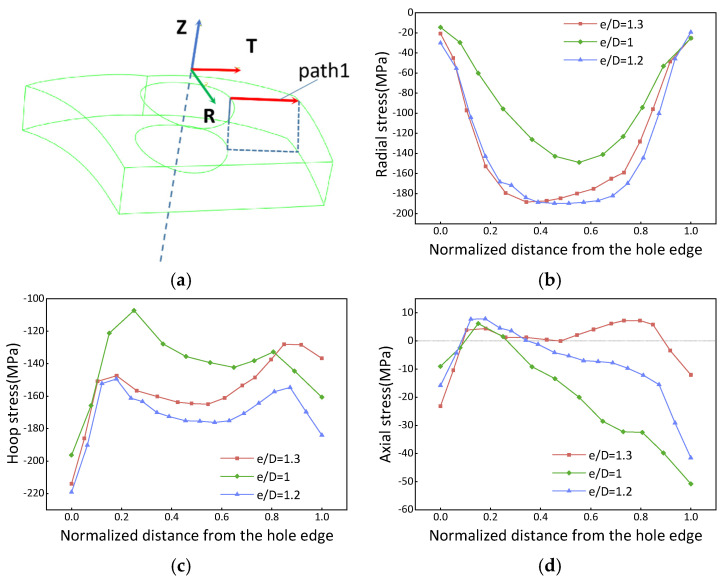
Surface residual stress after quenching. (**a**) Coordinate system and path; (**b**) radial stress; (**c**) hoop stress; (**d**) axial stress. The red arrow (path1) in subfigure (**a**) indicates the direction of the path.

**Figure 5:**
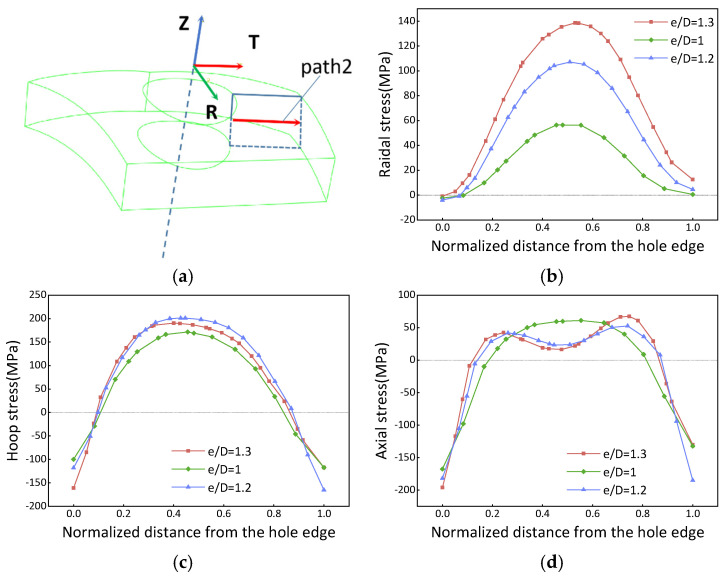
Internal residual stress distribution of the quenched forgings. (**a**) Coordinate system and path; (**b**) radial stress; (**c**) hoop stress; (**d**) axial stress. The red arrow (path2) in subfigure (**a**) indicates the direction of the path.

Figure 3e demonstrates the internal stress distribution, which showcases a layer-by-layer pattern in the quenched state. The temperature inconsistencies experienced during the quenching process result in a gradual decrease in hardness from the material’s surface to its center. As a result, the distribution of these stresses exhibits an almost symmetric pattern across the cross-section. Further details regarding this distribution can be found in the subsequent sections, specifically in Figure 4 and Figure 5.

#### 3.1.2. Surface Residual Stress

The stress distribution on the surface of sections A, B, and E in Figure 3e, considering different edge distance ratios (e/D = 1, 1.2, and 1.3), is shown in Figure 4. It is observed that the stress magnitude is higher in the middle region along the path from the hole edge to the free edge. Furthermore, the peak residual stress noticeably increases as the e/D increases from 1 to 1.2. However, beyond e/D = 1.2, the residual stress magnitude remains essentially constant. The behavior of the residual stress magnitude is influenced by temperature gradients when e/D is smaller than 1.2. In this range, the quenched stress increases as the e/D ratio increases during the quenching process. On the other hand, when the e/D ratio exceeds 1.2, the stress becomes limited by the quenching yield strength, resulting in the maximum residual stress being reached. In terms of the stress components, the axial component stress exhibits a lower magnitude than other components, typically ranging from −50 MPa to 10 MPa.

#### 3.1.3. Internal Residual Stress

Figure 5 depicts the internal stress distribution map of the PCG. The internal stress exhibits a symmetrical distribution along the center of the path. In this section, the magnitudes of the quenched yield strength are approximately 200 MPa. The hoop component stress is controlled by the thermal gradient when the e/D ratio is smaller than 1.2. However, beyond this threshold, it becomes limited by the quenched yield strength. On the other hand, the radial stress increases as the e/D ratio decreases. The peak value of the axial stress is approximately 50 MPa at the center, and its distribution transitions from an inverted U-shaped curve to an M-shaped curve when the e/D ratio exceeds 1. This paper only focuses on the stress distribution within the PCG, and no further detailed information regarding the e/D ratio and quenching medium temperature issues is provided. 

Regions located farther away from the quenching center experience a slower egtheld, than magnitudes of the quenched yield strength are heat absorption rate, resulting in larger temperature gradients and thus higher residual stresses. Conversely, regions closer to the quenching center absorb heat more rapidly, leading to smaller temperature gradients and lower residual stresses. This pattern becomes critical when the e/D ratio reaches 1.2. For e/D values greater than 1.2, the peak residual stress is limited by the quenched yield strength. Conversely, when the e/D ratio is lower than 1.2, the stress is primarily controlled by the thermal gradient. 

### 3.2. Bulging Residual Stress

Utilizing an FE model with a 1% bulging ratio, the detailed calculation results of stress reduction are shown in the following content.

#### 3.2.1. Overall Residual Stress Distribution

**Figure 6:**
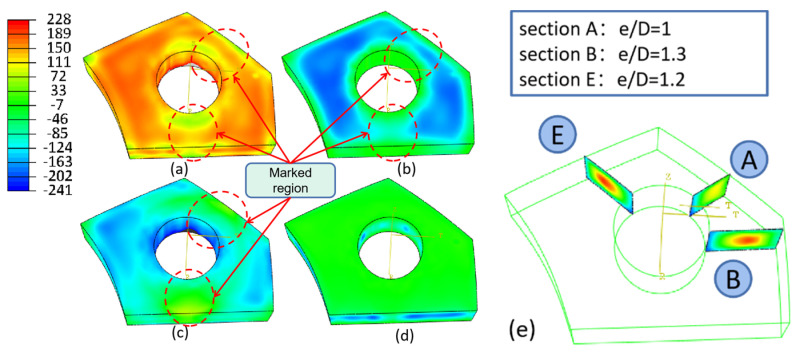
Residual stress distribution after the bulging process. (**a**) Mises stress; (**b**) radial stress; (**c**) hoop stress; (**d**) axial stress; (**e**) internal hoop stress.

**Figure 7:**
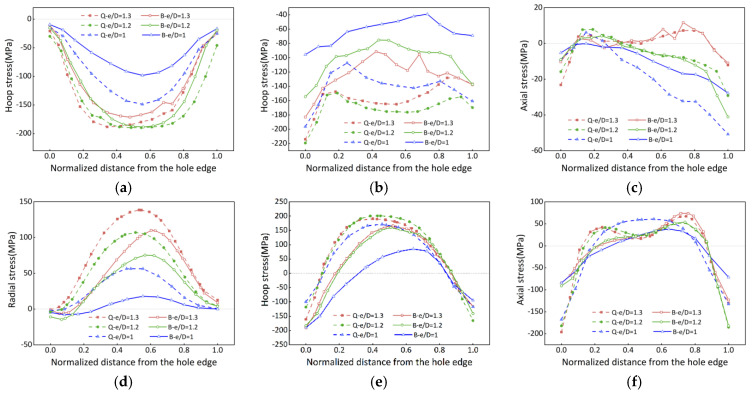
Surface and internal stress reduction in the bulging process. (**a**) Radial stress; (**b**) hoop stress; (**c**) axial stress; (**d**) radial stress; (**e**) hoop stress; (**f**) axial stress. Where (**a**–**c**) represent the surface radial, hoop, and axial components of stress, respectively; and (**d**–**f**) represent the internal radial, hoop, and axial components of stress, respectively.

Figure 6 depicts the stress contour map of the PCG after the bulging process. A significant reduction in residual stress is observed compared to Figure 3. Based on the previous analysis, the hoop component stress has the most substantial impact on the magnitude of the Mises stress, and it undergoes the most significant stress reduction during the bulging process. However, there is an increase in hoop stress on the inner side of the hole where it contacts the bulging block. This increase is beneficial for the material’s properties due to work hardening. Stress reduction is more pronounced in regions with smaller e/D values (marked in Figure 6), particularly at the hole edge. The surfaces that are parallel to the axis orientation exhibit remarkable stress reduction, while the stress levels remain constant on the top and lower surfaces. Stress reduction is more effective in sections with a smaller e/D ratio. Further details are illustrated in Figure 7.

#### 3.2.2. Evolution of Stress after Bulging

A formula was defined to quantify the reduction stress of the bulging process. The average stress reduction is given by:

(2) $$R_{a}=100\%\times \left\{\sum_{i=1}^{n}\left(\left|\sigma_{Q}\right|-\left|\sigma_{B}\right|\right)/\left|\sigma_{Q}\right|\right\}/n$$

where $\sigma_{Q}$ represents the quenched residual stress and $\sigma_{B}$ denotes the residual stress after bulging.

Figure 7a–c provides a detailed illustration of the surface stress evolution during the bulging process of the PCG. The degree of stress reduction is highly dependent on the e/D values, with greater stress reduction observed as the e/D value increases. The overall stress reduction follows a two-stage variation pattern. During the first stage, stress reduction gradually increases, but it decreases during the second stage. The axial component orientation undergoes only minimal plastic deformation during bulging, resulting in negligible stress reduction. Figure 7d–f demonstrates the internal stress distribution during the bulging process. It is evident from Figure 7 that the quenched residual stress transitions from tension stress to compression stress. This reversal may be attributed to compression occurring in the interior while the surface undergoes stretching. As a result, Equation (2) is no longer suitable for calculating stress reduction. Instead, the peak stress relief method is employed to assess internal stress reduction. The peak stress refers to the maximum tensile stress (positive value) in the central region, and the peak stress reduction can be calculated as:

(3) $$R_{p}=100\%\times \left\{\sigma_{Q,p}-\sigma_{B,p}\right\}/\sigma_{Q,p}$$

Surface stress reduction is determined using Equation (2), while internal stress reduction is calculated using Equation (3). The results are summarized in Table 3. It has been observed that stress reduction increases as the e/D value decreases for both surface stress and internal stress cases. Surface stress reduction is more effective during the bulging process (as it represents an average value), while internal stress reduction represents the peak value. However, significant deviations in the calculated results are observed for the axial component stress. This discrepancy arises due to the small axial component stress induced by the quenching process, which exhibits minimal variation in magnitude, but experiences significant fluctuations after bulging. Therefore, in subsequent sections, the axial component stress can be neglected to reduce the error in assessing the overall stress reduction.

The results of the bulging process confirm its effectiveness in reducing residual stress. This mechanical method combines tension in the hoop component orientation and compression in the radial component orientation to achieve stress reduction. Notably, the reduction of stress in the hoop component is significantly more effective than in the radial stress, potentially due to increased frictional effects at the ends during compression [15]. Furthermore, there is a variation in stress along the path, starting from the region near the hole edge and ending near the free edge. This variation arises from the different loading histories experienced. At the beginning of the path, stress reduction occurs through plastic deformation, followed by a hardening process during loading. However, the stress reduction is limited by the yield stress after unloading. At the end of the path, stress reduction is achieved through elastic deformation to facilitate stress redistribution after unloading. Moreover, regions with small e/D values exhibit lower stiffness, making them more prone to plastic deformation. Consequently, these regions experience more significant stress reduction during the bulging process. Increasing the bulging ratio leads to a substantial reduction in stress. Therefore, in the subsequent discussion, the CE (bulging ratio) was chosen as the parameter to investigate stress reduction. 

### 3.3. Assessment of the Overall Stress Reduction

**Figure 8:**
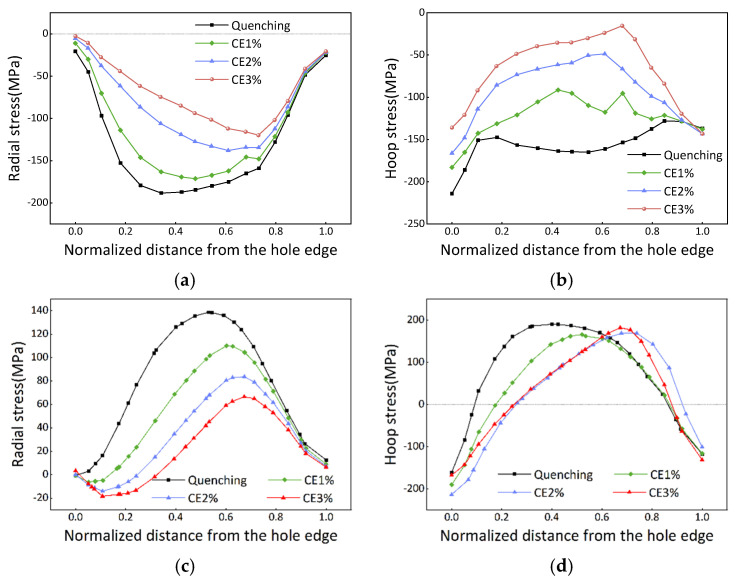
Residual stress with different bulging ratios after bulging. (**a**,**b**) Surface stress; (**c**,**d**) internal stress.

A comparative analysis was conducted to examine the effect of different bulging ratios on the overall stress reduction in the PCG. Figure 8 depicts the redistribution of stress on the B section with an e/D of 1.3. The results clearly demonstrate that stress reduction increases as the bulging ratio, represented by the CE, increases. Both internal stress and surface stress reduction exhibit a two-stage variation pattern, characterized by an initial increase followed by a subsequent decrease. 

A weighting function-based assessment method was established to evaluate the overall stress reduction in the PCG. First, three different regions with the e/D values of 1, 1.2, and 1.3 were selected as indexes, and the objective function was defined as the overall stress reduction. Second, the indexes were linearly weighted and incorporated into the objective function, which can be expressed as follows:

(4) $$Y=aX_{1}+bX_{2}+cX_{3}$$

Here, Y represents the objective function; $X_{1}$ represents the average stress reduction of the region with e/D = 1; $X_{2}$ denotes the average stress reduction of the region with e/D = 1.2; $X_{3}$ is the average stress reduction of the region with e/D = 1.3, *a*, *b*, and *c* are the weight coefficients. The weight coefficients *a*, *b*, and *c* satisfy the relationship:

(5) $$\left|a\right|+\left|b\right|+\left|c\right|=1$$

The weighting principles in this case are as follows: (1) Regions with greater e/D values are more challenging to reduce stress, so their weights should be relatively higher. (2) Taking into account the geometric characteristics, the proportions of regions with the e/D values = 1, 1.2, and 1.3 are 40%, 40%, and 20%, respectively. The higher the proportion, the greater the weight. Therefore, the weight coefficients assigned to each index are as follows: *a* = 0.3, *b* = 0.4, *c* = 0.3. 

Table 4 summarizes the data of residual stress reduction in the PCG. As the e/D value increases, the overall stress reduction also increases. When the CE(%) is increased from 1% to 2%, the radial component stress reduction increases by 19.7%, and the hoop component stress reduction increases by 14.6%. Similarly, when the CE is increased from 2% to 3%, the stress reduction increases by 11% and 5% for the radial and hoop components, respectively. Moreover, in the case of e/D = 1, the hoop component stress reduces with an increase in the bulging ratio. This observation can be explained by the reversal of compressive surface-quenched stress as the bulging ratio increases, as calculated using Equation (3). Accordingly, when the CE = 3%, tensile residual stress with a peak value of 151 MPa is observed in regions with e/D = 1, and the Mises stress tends to increase. The increased Mises stress can lead to subsequent machining distortion. Considering both the contribution of stress reduction and the detrimental factor, a CE(%) of 2% is determined to be the most suitable process parameter for bulging. 

### 3.4. Experiment Validation

#### 3.4.1. Experiments

**Figure 9:**
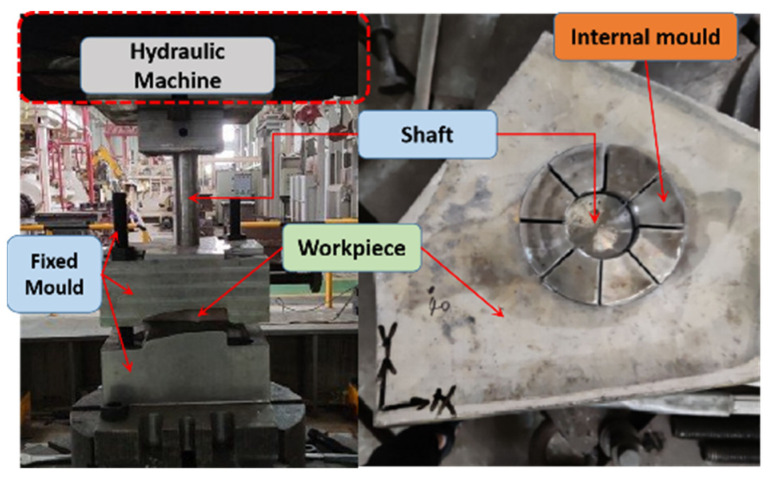
Bulging experiment.

Two workpieces underwent a solution heat treatment (SHT) process. The SHT conditions involved heating the workpieces to 477 °C for a duration of 600 min, followed by rapid quenching in water at 20 °C. Subsequently, the bulging process was conducted using a 4000-ton hydraulic machine, with a bulging ratio (CE) set at 2%. The bulging process is illustrated in Figure 9. 

#### 3.4.2. X-ray Diffraction Residual Stress Measurement

The surface residual stress was evaluated using an X-ray diffractometer. To minimize the influence of natural aging on residual stress, the measurements were performed within 2 h after the quenching process. Prior to the measurements, the designated surface was subjected to electropolishing to reduce any errors arising from poor surface quality. The X-ray diffraction parameters utilized for the measurements are provided in Table 5. 

#### 3.4.3. The Contour Method Stress Measurement

The contour method (CM), developed by Prime [26], was used to measure the internal residual stress in the PCG. This destructive testing technique offers a distinct advantage by providing a two-dimensional map of residual stress across a specific plane with a relatively high spatial resolution. Extensive research on the application of the contour method has demonstrated a strong correlation between the test results and those obtained through neutron diffraction [27,28,29]. The CM procedure involves the following main steps: (1) utilizing electro-discharging machining (EDM) to cut the parts with a copper wire; (2) determining the surface displacement by employing a coordinate measuring machine (CMM); and (3) smoothing and averaging the obtained contour test data, importing it into the ABAQUS FE software (version 6.14), and calculating the stress using elasticity principles. For mapping the cross-section contour, a regular grid of 3 mm × 3 mm was employed. The Young’s modulus was set to 71,500 GPa and the Poisson’s ratio was assigned a value of 0.33. 

#### 3.4.4. Experiment Results

**Figure 10:**
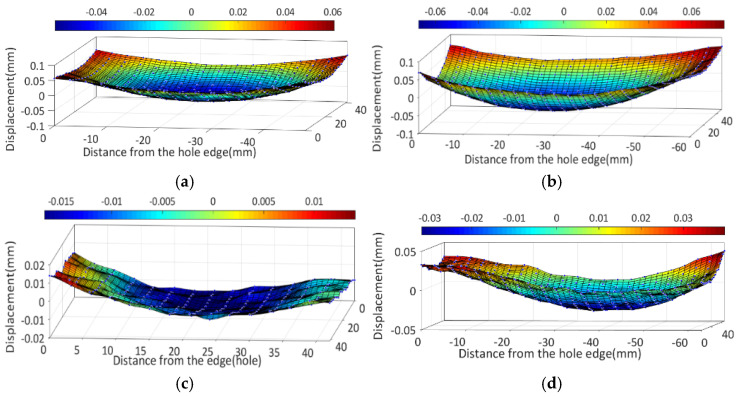
Contour maps of sections of the PCG in different process states. (**a**) e/D = 1 in the quenched state; (**b**) e/D = 1.2 in the quenched state; (**c**) e/D = 1 in the bulging state; (**d**) e/D = 1.2 in the bulging state.

Figure 10 demonstrates the contour map depicting the PCG in various process states. The section with e/D = 1.2 exhibits a maximum normal displacement of 0.068 mm and −0.075 mm in the quenched state, which subsequently reduces to 0.032 mm and −0.04 mm after bulging, respectively. Similarly, the quenched state yields maximum and minimum displacement values of 0.061 mm and −0.058 mm, respectively, for the section with an e/D = 1, which decrease to 0.014 mm and −0.017 mm, respectively, after bulging. The normal displacement of the section refers to the elastic deformation generated by stress release after cutting. The substantial disparity in displacement between the quenched and bulging processes signifies a significant reduction in stress during the bulging process.

**Figure 11:**
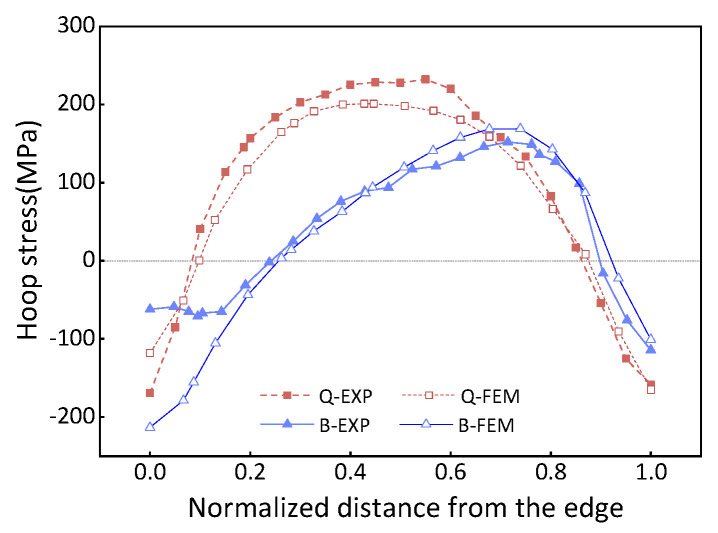
Comparison of internal residual stresses, where Q-FEM represents quenched stress in the FEM model; B-FEM represents bulging stress in the FEM model; Q-EXP denotes quenched stress determined by the contour method; B-EXP shows bulging stress determined by the contour method.

Figure 11 presents a comparison between the determined stress and the results obtained from FE simulation. It has been observed that there is a lack of fit at the contour edge, which can be attributed to the cutting sequence inducing rapidly varying boundary conditions near the hole edge. However, a satisfactory agreement is observed between the test results and the simulation. Therefore, the data from the hole edge are disregarded, resulting in a deviation of 13.2% between the experiments and FEM values. The deviation is given by:

(6) $$E\left(\%\right)=\frac{\left[\sum_{1}^{n}\left|\frac{\left(\sigma_{FEM,i}-\sigma_{EXP,i}\right)}{\sigma_{EXP,i}}\right|\right]}{n}\times 100\%$$

Here, $\sigma_{FEM,i}$ represents the stress simulation values at the *i*-th point along the path, and $\sigma_{EXP,i}$ denotes the measured stress values at the *i*-th point along the path.

**Figure 12:**
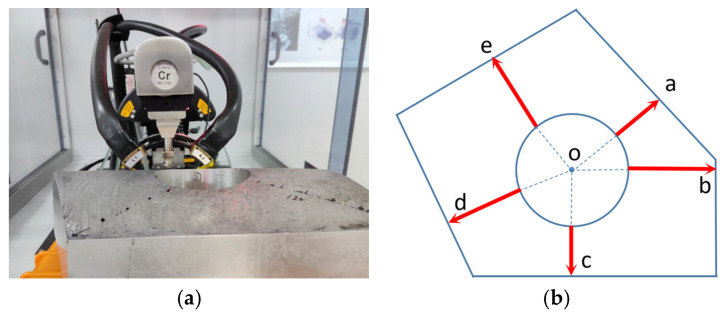
Surface residual stress measurement. (**a**) X-ray diffraction; (**b**) measurement path. The red arrow indicates the measurement direction of the path.

To obtain a comprehensive profile of cross-sectional stress distribution, the surface residual stress was determined using X-ray diffraction, as depicted in Figure 12. Due to the severe discrepancies observed at the section edge, this method was employed to overcome the limitations and acquire a complete stress distribution profile. To optimize the experimental time, only the hoop stress was determined in this particular study. 

**Figure 13:**
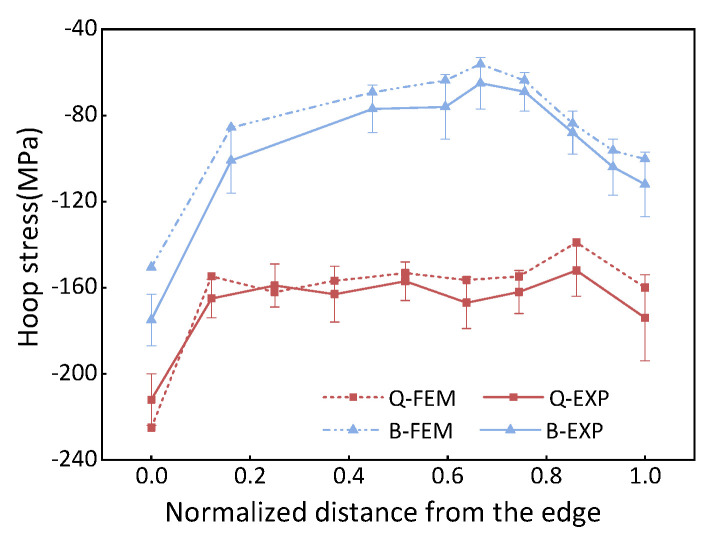
A comparison of residual stress measured in simulation and experiment at the quenched–bulging process.

Figure 13 shows the stress distribution measured by X-ray before and after bulging. The experimental results show that the quenched stress decreases by 44% after bulging. The deviation between the simulation and the experiment is determined to be 8%. 

The experimental results indicate that there is reasonable agreement between the experimental measurements and the simulations.

## 4. Conclusions

(1) The geometric features of the PCG were characterized based on the edge distance ratio (e/D). Three types of sections were identified: e/D = 1, 1.2, and 1.3.
(2) A thermo-mechanical FE model was developed to study the quenching process of the PCG. The analysis revealed that as the e/D ratio decreases, the stress amplitude increases. At e/D = 1.2, both hoop and radial component stresses reached peak values of approximately −200 MPa on the surface due to limitations in the quenching yield strength. Stresses at the hole edge and free edges are higher than those on the surface due to different heat transfer boundary conditions, with minimum values occurring at corners. Axial stresses on the upper and lower surfaces ranged from −50 MPa to 10 MPa, with maximum values concentrated in surfaces parallel to the axis orientation. Internally, the hoop stress reached its peak value at e/D = 1.2 (approximately 200 MPa), while the radial stress is relatively smaller, but increases with increasing e/D. When e/D exceeds 1, the axial stress distribution transitions from an inverted U-shaped curve to an M-shaped curve.
(3) An FE model for the bulging process was established to simulate stress reduction. Average stress reduction (*R_a_*) and peak stress reduction (*R_p_*) were used to calculate surface stress reduction and internal stress reduction, respectively. The results demonstrate that stress reduction increases as the e/D ratio decreases. Stress reduction exhibits a pattern of initial increase followed by a decrease along the path from the hole edge to the free edge. Furthermore, the results indicate that the bulging process is more effective in reducing surface stress than internal stress.
(4) Further simulations have been conducted to assess stress reduction under different bulging ratios (1%, 2%, 3%). An overall stress reduction evaluation was performed using a weight function based on different e/D ratios. The calculated results show that the overall stress reduction increases with the bulging ratio. Considering the contribution of stress reduction and the adverse factors of stress reversal, a bulging ratio of 2% is recommended as the most suitable for the PCG. The radial component stress reduction and hoop component stress reduction are determined to be 42.2% and 54.8%, respectively.
(5) X-ray diffraction and the contour method were employed to determine the surface and internal stresses of the PCG with CE = 2%. Comparative analysis of the measurement results with those obtained from the simulation revealed an 8% deviation in surface stress and a 13.2% deviation in internal stress. This indicates that the FE model for stress reduction during the bulging process exhibits good predictive accuracy.

## Figures and Tables

**Table 1:** The values of the e/D.

Series	Distance	e/D
A	98 mm	0.98
B	130 mm	1.3
C	97 mm	0.97
D	121 mm	1.21
E	119 mm	1.19

**Table 2:** FEM parameters for the quenching process.

Temperature °C	Conductivity w/m·°C	Elastic Modulus MPa	Yield Stress MPa	Specific Heat Capacity J/kg·°C	Density Kg/m^3^	Thermal Expansion m/°C
25	112	71,500	286	788	2730	3.00 × 10^−5^
50	116	69,460	245	852	2730	
100	126	66,830	218	925	2720	
150	137	64,100	199	937	2710	
200	155	61,420	162	861	2700	
250	168	58,580	146	938	2680	
300	168	55,330	94	1013	2670	
350	163	51,620	49	1053	2660	
400	158	49,960	39	1105	2650	
450	153	28,930	24	1113	2640	

**Table 3:** The results of the stress reduction with a bulging ratio of 1%.

	e/D	Radial Stress Reduction	Hoop Stress Reduction	Axial Stress Reduction
Surface stress	1	36%	56%	51%
	1.2	18%	41%	−42%
	1.3	15%	20%	−138%
Internal stress	1	69%	66%	37%
	1.2	28%	20%	28%
	1.3	20%	12%	20%

**Table 4:** The results of the overall stress reduction with different bulging ratios.

Component	CE 1% Stress Reduction		CE 2% Stress Reduction		CE 3% Stress Reduction	
	Radial Stress	Hoop Stress	Radial Stress	Hoop Stress	Radial Stress	Hoop Stress
$X_{1}$	36%	56%	77%	34%	86%	25%
$X_{1}$	18%	42%	23%	77%	34%	85%
$X_{1}$	15%	22%	33%	46%	46%	61%
Y	22.5%	40.2%	42.2%	54.8%	53.2%	59.8%

**Table 5:** X-ray diffraction parameters.

X-ray Diffraction Parameters	Values
X-ray diffractometer	XRD, STRESSTECH 3000
Tube type	Cr
Supplied current during the experiment	6.7 mA
Supplied voltage during the experiment	30 kV
Exposure time for the calibration	8 s
Exposure time for the measurement	10 s
Collimator diameter	2 mm
Collimator distance	10.5 mm
Tilt angle	−45°~45°
Number of tilts	5/5
Angle of deviation	139.7
Crystal face of deviation	313
Stress resolution	±10 MPa

## Data Availability

The data presented in this study are available on request from the corresponding author.
